# Regions important for the adhesin activity of *Moraxella catarrhalis *Hag

**DOI:** 10.1186/1471-2180-7-65

**Published:** 2007-07-03

**Authors:** Brian Bullard, Serena Lipski, Eric R Lafontaine

**Affiliations:** 1 Department of Medical Microbiology and Immunology, University of Toledo Health Sciences Campus, 3055 Arlington Avenue, Toledo, OH, 43614, USA; 2 Department of Infectious Diseases, University of Georgia College of Veterinary Medicine, Athens, GA, 30602, USA

## Abstract

**Background:**

The *Moraxella catarrhalis *Hag protein, an Oca autotransporter adhesin, has previously been shown to be important for adherence of this respiratory tract pathogen to human middle ear and A549 lung cells.

**Results:**

The present study demonstrates that adherence of *M. catarrhalis *isogenic *hag *mutant strains to the human epithelial cell lines Chang (conjunctival) and NCIH292 (lung) is reduced by 50–93%. Furthermore, expressing Hag in a heterologous *Escherichia coli *background substantially increased the adherence of recombinant bacteria to NCIH292 cells and murine type IV collagen. Hag did not, however, increase the attachment of *E. coli *to Chang cells. These results indicate that Hag directly mediates adherence to NCIH292 lung cells and collagen, but is not sufficient to confer binding to conjunctival monolayers. Several in-frame deletions were engineered within the *hag *gene of *M. catarrhalis *strain O35E and the resulting proteins were tested for their ability to mediate binding to NCIH292 monolayers, middle ear cells, and type IV collagen. These experiments revealed that epithelial cell and collagen binding properties are separable, and that residues 385–705 of this ~2,000 amino acid protein are important for adherence to middle ear and NCIH292 cells. The region of O35E-Hag encompassing aa 706 to 1194 was also found to be required for adherence to collagen. In contrast, β-roll repeats present in Hag, which are structural features conserved in several Oca adhesins and responsible for the adhesive properties of *Yersinia enterocolitica *YadA, are not important for Hag-mediated adherence.

**Conclusion:**

Hag is a major adherence factor for human cells derived from various anatomical sites relevant to pathogenesis by *M. catarrhalis *and its structure-function relationships differ from those of other, closely-related autotransporter proteins.

## Background

Autotransporter proteins form the largest known family of virulence factors expressed by Gram-negative bacteria, and play central roles in processes such as cell-to-cell aggregation, biofilm formation, serum resistance, and adherence to host epithelial cells [1]. The members of this family share structural features that include a surface-exposed passenger domain specifying the biological activity of the protein and a C-terminal transporter module comprised of several β-strands that anchors the autotransporter in the outer membrane (OM). Based on the length and structure of this transporter module, autotransporters can be classified as conventional (long module containing 12 β-strands) or trimeric (short module containing 4 β-strands) [2]. One of the better characterized trimeric autotransporter molecules is the *Yersinia enterocolitica *adhesin YadA; YadA and structurally related autotransporters are also often referred to as Oca (**O**ligomeric **c**oiled-coil **a**dhesin) proteins [3]. Because of their structure and role in pathogenesis, autotransporters represent promising targets for anti-infective approaches. Large portions of these proteins (the passenger domains) are located on the bacterial surface and are therefore amenable to recognition by the immune system. In addition, autotransporters play key roles in virulence and targeting them may interfere with development of disease.

*Moraxella catarrhalis *is a Gram-negative bacterium that causes a wide range of infections, including ~20% of all cases of bacterial otitis media in children [4], up to 10% of lung infections in elderly patients with Chronic Obstructive Pulmonary Disease (COPD) [5], sinusitis [6], and conjunctivitis [7]. This organism is a significant health concern, and complicating this problem is the fact that most *M. catarrhalis *clinical isolates display resistance to antibiotics including β-lactams [8-10]. The development of a vaccine for *M. catarrhalis *thus has increasing importance for the health status of both the elderly and the very young. This bacterium expresses several autotransporter proteins that have been well-studied including the trimeric/Oca adhesins UspA1 [3,11-18], UspA2H [11], and Hag/MID [13,15,19-26], the trimeric/Oca serum resistant factor UspA2 [3,11,16,17,27,28], and the conventional autotransporter adhesin/phospholipase B McaP [29,30].

Expression of Hag, or its ortholog MID (**M**oraxella **I**g**D**-binding protein), is important for attachment to erythrocytes as well as A549 (lung pneumocytes) and HMEE (human middle ear epithelial) cells [22-24,26], and Hag directly mediates adherence to HMEE cells [26]. In addition, Hag binds immunoglobulin D and forms oligomers [13,19,21,22,24]. The gene coding for this protein is found in almost all *M. catarrhalis *isolates studied to date [24,26], and specifies a relatively well-conserved molecule of ~2,000 residues in length. Expression of Hag is subject to translational phase variation, via slipped strand mispairing in a homopolymeric guanine tract located near the beginning of the ORF [13,24,26]. Hag has also been found to contain a series of degenerate amino acid (aa) repeats similar to those present in the N-terminus of the prototypical Oca adhesin YadA and which specify the adhesive properties of this *Y. enterocolitica *protein [26,31,32].

In this study, we sought to determine what role Hag plays in *M. catarrhalis *adherence to lung mucoepidermoid (NCIH292) and conjunctival (Chang) cells by measuring the binding of Hag isogenic mutants and of recombinant *E. coli *expressing wild-type (WT) *hag *genes to these human cells. We also sought to identify the region(s) of Hag important for adherence using a panel of in-frame *hag *deletion constructs.

## Results

### Hag expression is important for the adherence of *M. catarrhalis *to NCIH292 and Chang cells *in vitro*

Previous work demonstrated that Hag plays an important role in the attachment of *M. catarrhalis *to HMEE cells and A549 human type II pneumocytes [23,26]. To further explore the role of this protein in adherence to human epithelial cells that are relevant to pathogenesis by *M. catarrhalis*, a panel of previously-described WT isolates and cognate *hag *mutant strains [26] were tested for their ability to bind to NCIH292 lung mucoepidermoid and Chang conjunctival cells. Of note, these mutants were previously shown to have reduced adherence to A549 and HMEE cells [26].

**Figure 1:**
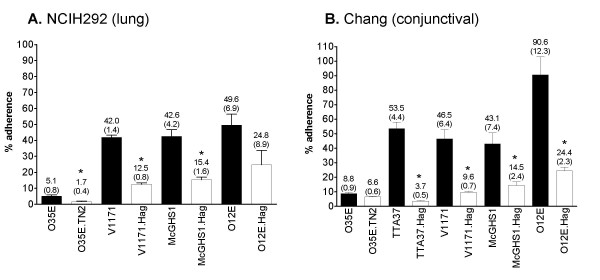
**Adherence of *M. catarrhalis*strains to NCIH292 (Panel A) and Chang (Panel B) cells**. Black bars correspond to WT isolates and *hag *isogenic mutant strains are represented by open bars. The results are expressed as the mean (± standard error) percentage of inoculated bacteria binding to monolayers. The number above each bar represents mean percentage; standard error is shown in parentheses. Asterisks indicate that the difference in adherence between a WT strain and its respective *hag *mutant is statistically significant.

Strain TTA37 attached poorly to NCIH292 monolayers and the adherence of its *hag *mutant, TTA37.Hag, was not significantly lower (data not shown). Hag-deficient strains derived from isolates V1171, O35E, McGHS1 and O12E did not attach as efficiently to NCIH292 cells as their respective WT progenitors (Fig. 1A). The binding of V1171.Hag, O35E.TN2 and McGHS1.Hag to these monolayers was about a third that of their parent strains V1171, O35E, and McGHS1, respectively. Though the two-fold difference between strains O12E and O12E.Hag was not statistically significant, the preponderance of our data suggests that Hag expression is important for the adherence of *M. catarrhalis *to NCIH292 lung cells.

Similar results were obtained when the WT and isogenic mutant strains were tested for their ability to bind to Chang conjunctival cells. The *hag *mutants of strains V1171, O12E and McGHS1 all adhered significantly less than their WT progenitors (Fig. 1B). Strain TTA37 attached well to Chang cells (unlike NCIH292 cells) and the adherence of its Hag deficient mutant TTA37.Hag was reduced by 93%. Strain O35E.TN2 did not show Hag-dependent binding to Chang cells, though strain O35E did not attach as well to these monolayers as the other WT *M. catarrhalis *isolates tested. Taken together, these results indicate that Hag expression is important for the binding of *M. catarrhalis *to conjunctival cells. Of note, all strains tested in this study were previously shown to express WT levels of the adhesins UspA2H (in the case of strain TTA37), UspA1, McaP, and OMPCD [26], yet we found that Hag deletions still substantially reduce adherence.

### Hag directly confers binding to NCIH292 cells and murine type IV collagen

**Figure 2:**
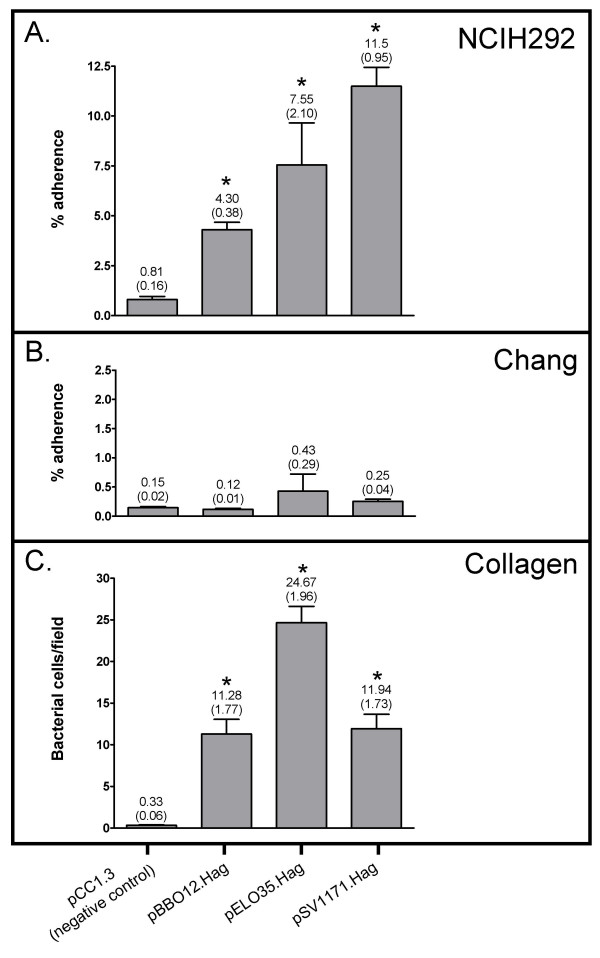
**Adherence of recombinant *E. coli *bacteria expressing WT Hag proteins**. Panels A and B: Adherence is expressed as the mean percentage (± standard error) of inoculated bacteria binding to monolayers. Panel C: Adherence is expressed as the normalized mean number of bacteria per microscopic fields (± standard error) binding to collagen type IV coated wells. The number above each bar represents the mean; standard error is shown in parentheses. Asterisks indicate that the difference in binding between *E. coli *expressing O35E-Hag (i.e. pELO35.Hag), O12E-Hag (i.e. pBBO12.Hag) or V1171-Hag (i.e. pSV1171.Hag) and the negative control is statistically significant.

It is possible that absence of Hag in the OM of *M. catarrhalis *mutants affected the proper display of other adhesins, which themselves mediate adherence to NCIH292 and Chang monolayers. To determine whether Hag directly mediates binding to these epithelial cells, recombinant *E. coli *strains previously shown to express the *hag *gene product of *M. catarrhalis *strain O12E, O35E or V1171 [26] were tested in quantitative attachment assays. Of note, these clones were previously shown to have gained the ability to attach to HMEE cells [26]. As shown in Fig. 2A, O12E-, O35E- and V1171-Hag conferred on *E.coli *the ability to bind to NCIH292 cells at levels that were ~5-, ~10- and ~15-fold greater than *E. coli *carrying the control plasmid pCC1.3, respectively. In contrast, *E. coli *expressing these three Hag proteins did not show significant increases in binding to Chang monolayers (Fig. 2B). These results demonstrate that Hag directly mediates adherence to NCIH292 cells, but its expression is not sufficient to increase the binding of recombinant *E. coli *to Chang monolayers.

Based on sequence and structural similarities, Hag belongs to the Oca family of autotransporter adhesins [26]. *Y. enterocolitica *YadA is the prototypical member of this family and has been shown to directly mediate adherence to different types of collagen [31-33], including type IV [33-35]. *E. coli *cells expressing the Hag protein of *M. catarrhalis *strain O35E, O12E or V1171 were therefore tested for their ability to bind to murine type IV collagen. As shown in Fig. 2C, *E. coli *expressing O12E- and V1171-Hag were ~35 times more adherent to collagen than the negative control while recombinant bacteria expressing O35E-Hag exhibited a ~80-fold increase in binding. These data indicate that Hag proteins from different *M. catarrhalis *isolates directly mediate attachment to murine type IV collagen when expressed in the *E. coli *background. However, our panel of *M. catarrhalis *WT and isogenic mutant strains all bound poorly to collagen-coated wells (data not shown).

### Construction and surface-expression of mutated Hag proteins

**Figure 3:**
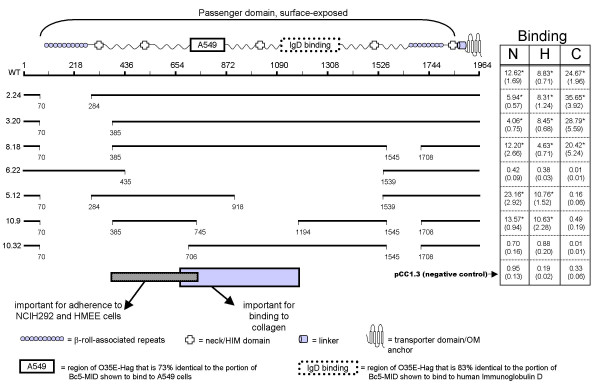
**Selected structural features of O35E-Hag, schematic representation of in-frame deletions introduced in the protein, and adherence of recombinant *E. coli *bacteria expressing mutated Hag**. Selected structural features are shown above in relation to their physical location within the O35E-Hag protein. Deletions within the protein are represented by gaps. Construct names are shown to the left. The columns to the right of each construct indicate the binding of *E. coli *expressing the mutated protein to NCIH292 cells (N), HMEE (H) or collagen (C). Adherence to NCIH292 and HMEE cells is expressed as the mean percentage (standard error shown in parentheses) of inoculated bacteria binding to monolayers. Adherence to collagen is expressed as the normalized mean number of bacteria per microscopic fields (standard error shown in parentheses) binding to collagen type IV coated wells; we estimated that 20 to 35 bacteria per microscopic field corresponds to 5–15% of input bacteria. Asterisks indicate that the difference in binding between *E. coli *expressing Hag proteins and the negative control is statistically significant. The negative control corresponds to *E. coli *carrying the plasmid control pCC1.3 and WT corresponds to recombinant bacteria harboring plasmid pELO35.Hag. The rectangles at the bottom indicate regions important for adherence to epithelial cells and collagen.

**Figure 4:**
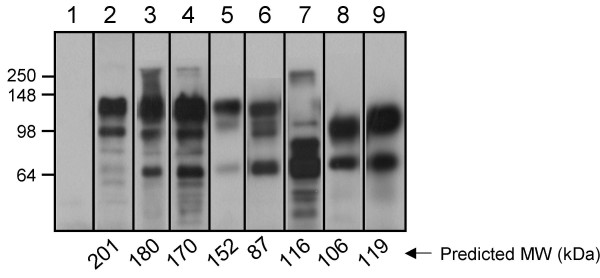
**Western blot analysis of Sarkosyl-insoluble OM proteins extracted from *E. coli *expressing Hag proteins**. OM preparations were obtained from *E. coli *carrying the plasmids pCC1.3 (lane 1), pELO35.Hag (lane 2), pBBHS2.24 (lane 3), pBBHS3.20 (lane 4), pBBHS8.18 (lane 5), pBBHS6.22 (lane 6), pBBHS5.12 (lane 7), pBBHS10.9 (lane 8) and pBBHS10.32 (lane 9). These preparations were resolved by SDS-PAGE, transferred to PVDF membranes and probed with antibodies against the purified recombinant protein His.Hag.CT. The figure is a composite of several western blot experiments in which OM preparations of the negative control (i.e. pCC1.3) and the positive control (i.e. pELO35.Hag) were included. Molecular weight markers are shown to the left in kDa. The numbers at the bottom of the western panel represent the predicted molecular weight of each Hag protein.

To define the region(s) of Hag important for adherence to epithelial cells and collagen, several in-frame deletions were engineered in the *hag *ORF of strain O35E (specified by the plasmid pELO35.Hag). Fig. 3 illustrates the various deletion constructs that were made. These mutated Hag proteins were tested for their ability to mediate the binding of recombinant *E. coli *to NCIH292 cells, HMEE cells and murine collagen (see below). Sarkosyl-insoluble OM proteins were also prepared from recombinant *E. coli *expressing these constructs, and tested by immunoblotting with Hag-reactive antibodies. As shown in Fig. 4, all mutated Hag proteins were associated with the OM. The polyclonal antibodies used in these westerns are against the last 607 residues of O35E-Hag (i.e. His.Hag.CT, see Methods) and encompass the transporter domain (Fig. 3). This portion of Oca adhesins has been shown to be important for oligomerization properties, which likely accounts for the multiple bands reacting with anti-Hag antibodies in Fig. 4.

**Figure 5:**
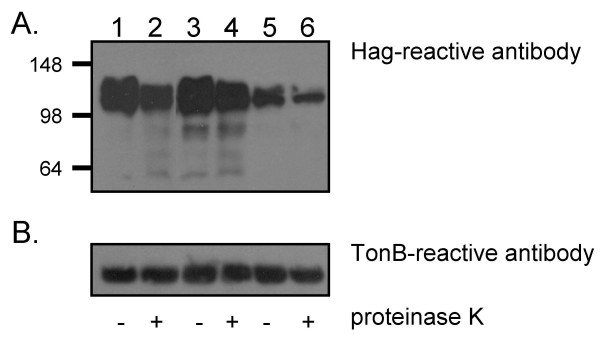
**Western blot analysis of recombinant *E. coli *cells treated with proteinase K**. *E. coli *carrying the plasmids pELO35.Hag (lanes 1 and 2), pBBHS2.24 (lanes 3 and 4) and pBBHS3.20 (lanes 5 and 6) were incubated for 15 min on ice in the presence (lanes 2, 4 and 6) or absence (lanes 1, 3 and 5) of proteinase K. These cells were lysed, resolved by SDS-PAGE, transferred to PVDF membranes and probed with the anti-Hag antibody 5D2 (panel A) or anti-TonB antibody 4F1 (panel B). Molecular weight markers are shown to the left in kDa.

To verify that these OM-located Hag proteins were exposed on the bacterial surface, intact recombinant *E. coli *cells were treated with proteinase K and analyzed by Western blot using antibodies against Hag. As controls, proteinase K-treated bacteria were also probed with a monoclonal antibody against the inner membrane-anchored protein TonB [36] to demonstrate that a minimal amount of the enzyme transversed across the OM and into the periplasm of *E. coli*. Fig. 5 shows the results of a representative experiment in which *E. coli *carrying the plasmids pELO35.Hag, pBBHS2.24 and pBBHS3.20 were incubated for 15 min on ice in the absence (lanes 1, 3 and 5) or presence (lanes 2, 4 and 6) of proteinase K. As shown in Fig. 5A, incubation with the enzyme decreases the reactivity of the Hag-specific monoclonal antibody 5D2 to the cell lysates. By contrast, little to no degradation of TonB was observed under these conditions (Fig. 5B). Longer incubation with proteinase K resulted in the near-complete digestion of Hag but also caused partial degradation of TonB (data not shown). The data are therefore consistent with the Hag proteins being exposed on the surface of recombinant bacteria (personal communication from Dr. Ray Larsen, Bowling Green State University); similar results were obtained when *E. coli *expressing the other mutated Hag proteins were treated with this enzyme (data not shown).

### Adhesive properties of recombinant Hag proteins containing in-frame deletions

As previously reported [26], one striking similarity between the *Y. enterocolitica *adhesin YadA and O35E-Hag is the presence of degenerate aa sequence repeats in their N-termini, which in YadA form coiled parallel structures described as a β-roll [31]. O35E-Hag contains 10 such N-terminal repeats between residues 85–274 (see Fig. 3, top) having the consensus sequence GxxSIAIGxx(A/S)xAx. For comparison, the YadA β-roll repeats are xxxSVAIGxxSxAx [26]. In this *Y. enterocolitica *adhesin, the repeated motifs are followed by a "neck" region (HIM domain, Pfam accession # PF05662), which is also present in O35E-Hag after the β-roll repeats (aa 312–336 in Fig. 3). Of significance, the β-roll specifies the adhesive properties of YadA, and an intact neck region appears to be essential for the proper display/stability of the β-roll since mutations in either area abolishes YadA-dependent binding to human cells and collagen [31-35]. Based on these observations, we hypothesized that the β-roll and neck region located in the N-terminus of O35E-Hag are responsible for epithelial cell and/or collagen binding functions. To test this hypothesis, the region of Hag encompassing only the β-roll repeats (aa 71–283, construct 2.24 in Fig. 3) or the β-roll structure and its associated neck (aa 71–384, construct 3.20) were deleted. As shown in columns H and C of Fig. 3, these mutations did not affect binding to HMEE cells or collagen, respectively. In addition, *E. coli *cells expressing these two deletion constructs attached to NCIH292 monolayers well above background levels (Fig. 3, column N). These results oppose the hypothesis that the N-terminal β-roll and associated neck region of O35E-Hag specify adhesive properties.

Unlike YadA, which contains only one β-roll/neck combination, O35E-Hag contains a second neck region in its C-terminus (aa 1842–1865 in Fig. 3) preceded by a β-roll structure containing 8 "GxxSIAIGxx(A/S)xAx" repeats (residues 1680–1798 in Fig. 3). This second β-roll/neck combination of O35E-Hag is located prior to a predicted membrane anchoring module and is presumably located on the bacterial surface as part of Hag's autotransporter passenger domain. To determine whether this C-terminal β-roll structure specifies adhesive properties, residues 1546–1707 (encompassing 2 "GxxSIAIGxx(A/S)xAx" motifs) were removed in construct 8.18, which also contains the deletion of the N-terminal β-roll/neck area (Fig. 3). Expression of this doubly-deleted Hag protein still conferred on *E. coli *cells the ability to bind to HMEE cells, collagen and lung cells at or near WT levels (columns N, H and C of Fig. 3). By contrast, recombinant bacteria expressing the mutated adhesin construct 6.22, which retains both β-roll/neck combinations but is missing residues 436–1538 (Fig. 3), no longer adhere to either epithelial cells or collagen (columns N, H and C of Fig. 3). These results therefore support rejection of the hypothesis that the β-roll structures of O35E-Hag specify adhesive properties as they do in YadA. Our data also suggest that residues 385–1545 encompass the collagen and epithelial cell binding domain(s) of this *M. catarrhalis *adhesin.

The *M. catarrhalis *strain Bc5 protein MID, a Hag ortholog, binds human immunoglobulin D on the bacterial surface, and MID residues 962–1200 are responsible for this binding [19,21,24]. Sequence analysis revealed that the region of O35E-Hag encompassing aa 1113–1353 is 83% identical to the Bc5-MID IgD binding domain (not shown). Furthermore, Hag mediates the binding of IgD by *M. catarrhalis *strain O35E [13]. Thus, the possibility that the IgD-binding domain of O35E-Hag is responsible for adherence to human epithelial cells and collagen was tested by removing aa 919–1538 (dotted rectangle labeled "IgD binding" at the top of Fig. 3) from the already mutated Hag protein 2.24, yielding the construct 5.12 (see Fig. 3). As shown in Fig. 3, deleting the predicted IgD binding region of O35E-Hag does not adversely affect attachment to epithelial cells (columns N and H) but completely abolishes binding to collagen (column C). Adherence to human cells is therefore specified by a region of O35E-Hag distinct from that important for binding collagen, making these adhesive properties separable.

The adhesive properties of constructs 8.18 and 5.12 suggest that residues 385–918 contain the portion of O35E-Hag mediating adherence to NCIH292 cells and HMEE cells. Interestingly, previous sequence analysis by our laboratory indicated that aa 715–863 of O35E-Hag are 73% identical to residues 764–913 of *M. catarrhalis* Bc5 MID [23]. This is noteworthy because others showed that a purified and radioactively-labeled recombinant protein encompassing this particular portion of Bc5 MID directly binds to A549 cells and erythrocytes [22]. To determine what role, if any, these aa have in adherence to NCIH292 cells, HMEE cells and collagen, most of the potential A549/erythrocyte binding site of O35E-Hag (rectangle labeled "A549"at the top of Fig. 3) was removed from the already mutated Hag protein 8.18, yielding construct 10.9 (see Fig. 3). As shown in columns N and H of Fig. 3, *E. coli* expressing this construct attached to both NCIH292 lung cells and HMEE cells at the same level as recombinant bacteria expressing the full length adhesin. However, these recombinant bacteria, like those expressing 5.12, no longer bound to collagen (column C of Fig. 3). Thus, the results are consistent with residues 385–745 encompassing a portion of O35E-Hag that is crucial for adherence to NCIH292 monolayers and HMEE cells. This hypothesis is also supported by the results of quantitative attachment assays with construct 10.32, which was obtained by deleting aa 385–705 from the adherence positive Hag construct 8.18 (see Fig. 3); recombinant *E. coli* expressing construct 10.32 no longer adhere to either NCIH292 or HMEE cells (Fig. 3). The inability of constructs 5.12, 10.9 and 10.32 to mediate the binding of recombinant bacteria to collagen suggests that a region of O35E-Hag starting upstream of aa 706 and extending to at least residue 1194 is important for adherence to this extracellular matrix protein (Fig. 3).

## Discussion

Previous reports established that Hag and its ortholog MID play important roles in adherence of *M. catarrhalis *to middle ear epithelial cells and A549 pneumocytes [22,23,26]. Disruption of *hag/mid *in several *M. catarrhalis *strains substantially decreased adherence to both cell types, while expression of *hag *in a heterologous *E. coli *background increased attachment to middle ear cells at least 17-fold. The results of the present study extend these findings and demonstrate that Hag expression is also important for adherence to conjunctival (Chang) and lung mucoepidermoid (NCIH292) epithelial cells (Fig. 1). These cell lines are relevant to pathogenesis by *M. catarrhalis *as this organism is a causative agent of conjunctivitis, otitis media and lung infections. Furthermore, our data reveal that Hag directly mediates binding to NCIH292 monolayers (Fig. 2A). Thus, Hag is a key adherence factor for epithelial cells relevant to pathogenesis by the bacterium, making the protein an attractive target for anti-infective approaches.

Hag/MID exhibit several properties of a good vaccine candidate. Most *M. catarrhalis *isolates tested to date contain a *hag/mid *gene and express its product [13,15,22,24,26]. The protein also contains surface-exposed epitopes [13,22,24], making it readily available for recognition by the immune system, and immunization with a peptide encompassing MID residues 713–964 increases clearance of *M. catarrhalis *from the lungs of infected mice [25]. Our data demonstrating that Hag plays a central role in *M. catarrhalis *adherence, a crucial step in pathogenesis by most infectious agents [37-39], strengthen the hypothesis that this OM protein has vaccinogenic potential. We believe that targeting functionally relevant regions of Hag in a vaccine will interfere with colonization of susceptible individuals by *M. catarrhalis*. Bacterial adhesins have already proven to be efficacious vaccine antigens. For example, immunization with the cell-binding domain of the *H. influenzae *adhesin Hap elicits production of antibodies that reduce attachment to human epithelial cells and protect mice in a nasopharyngeal colonization model [40]. Similarly, immunization with the adhesin FimH of uropathogenic *E. coli *is protective in animal models of infection [41,42]. Furthermore, all vaccines licensed for use in the US against whooping cough contain the *Bordetella pertussis *filamentous hemagglutinin adhesin FHA [43].

Also consistent with the vaccinogenic potential of Hag is the demonstration that saliva collected from healthy adults [44] and children colonized with *M. catarrhalis *[45] contain antibodies against Hag. COPD patients recovering from pulmonary exacerbations caused by this organism were also shown to have increased serum levels of anti-Hag IgG [17,46]. In addition, Hag is a major target of new IgA antibodies purified from the sputum of COPD patients with *M. catarrhalis *infections who have successfully cleared the organism [16]. In fact, Hag is the only OM protein out of several tested (i.e. UspA1, UspA2, CopB, TbpB) which elicited an IgA response in all patients [16]. This protective immune response, however, appears to be strain specific as COPD patients get reinfected with different *M. catarrhalis *isolates [5]. Hag is a large protein (1964–2335 aa) that exhibits interstrain variability (57–89% identity) [24,26]. These observations emphasize the need to identify the region(s) of Hag specifying the best vaccinogenic properties for protection against the majority of *M. catarrhalis *isolates. To achieve this, we sought to identify the region(s) of Hag specifying adhesive properties by deleting predicted structural domains of the protein. This approach has been successfully used to identify the cell-binding domains of other autotransporter adhesins such as *Y. enterocolitica *YadA [32] and *H. influenzae *Hap [47].

The involvement of Hag in adherence by *M. catarrhalis *is complex. While the protein itself is sufficient to directly mediate binding to middle ear [26] and NCIH292 lung cells (Fig. 2A), the exact role of Hag in attachment to A549 and Chang cells is not clear. As shown in Fig. 1B, lack of Hag expression in strains TTA37.Hag, V1171.Hag, McGHS1.Hag and O12E.Hag reduces binding to Chang monolayers 70–90%, but Hag expression does not confer on *E. coli *the ability to attach to these conjunctival cells (Fig. 2B). These results are similar to those previously reported by our laboratory, where it was discovered that Hag expression is important for WT adherence of *M. catarrhalis *to A549 cells but that expression of the protein is not sufficient to mediate attachment of recombinant *E. coli *cells to these pneumocytes [26]. Complicating matters is the demonstration that purified Bc5-MID, specifically residues 713–964, binds to A549 cells [22]. These observations suggest that post-translational modification of the protein and/or expression of a co-adhesin, neither of which occurs in the heterologous *E. coli *background, is necessary for Hag-dependent binding to A549 and Chang monolayers. Interestingly, lack of OMPCD [48] or UspA1 [11,12] substantially decreases adherence of *M. catarrhalis *to A549 and Chang cells, respectively. These molecules may thus act in concert with Hag during attachment to these epithelial cells. The lack of a suitable plasmid vector for *M. catarrhalis *currently precludes us from directly addressing these issues in the native genetic background. Only three plasmids, namely pWW102B [49], pWW115 [50] and pEMCJH04 [51], have been described for use in *M. catarrhalis*. These vectors are not amenable for complemention of our mutants due to the incompatibility of antibiotic^R ^markers and replication in a restricted number of *M. catarrhalis *isolates. For these reasons, we focused our efforts on identifying region(s) of O35E-Hag important for directly mediating adherence to HMEE and NCIH292 cells, using *E. coli *as a host for our experiments.

The adhesive properties of constructs 5.12, 8.18 and 10.9 imply that residues 71–384 and 746–1707 of O35E-Hag do not specify binding to either HMEE or NCIH292 monolayers (Fig. 3). These results are particularly interesting in light of previous reports that the β-roll repeats of YadA specify binding to both collagen and human epithelial cells [31-35]. A recent study also indicates that the β-roll repeats found in the N-terminus of the *Actinobacillus actinomycetemcomicans *Oca autotransporter adhesin EmaA specify adhesive properties [52]. The ability of construct 8.18 to confer on *E. coli *the ability to attach to NCIH292 cells, HMEE cells and collagen, however, demonstrates that the β-roll structures of O35E-Hag are not required for adherence. Although all mutated Hag proteins conferring attachment to epithelial cells also have aa 1–70 and 1708–1964 in common (Fig. 3), it is unlikely that these residues are directly mediating adherence. This belief is based on previous sequence analysis of O35E-Hag indicating that aa 1–66 correspond to a signal sequence and that the last 100 residues (i.e. 1864–1964) specify a transporter domain anchoring the protein in the OM [26]. Furthermore, recombinant bacteria expressing the mutated Hag proteins 6.22 and 10.32, both of which specify aa 1–70 as well as 1708–1964 but lack most of residues 385–745, do not bind to HMEE or NCIH292 cells (Fig. 3). These observations support the hypothesis that aa 385–745 of O35E-Hag contain a major epithelial cell binding determinant.

Forsgren and colleagues previously reported that residues 764–913 of MID, which correspond to aa 715–863 of O35E-Hag, encompass the part of the protein mediating adherence to A549 cells and erythrocytes [22]. Our results demonstrate that the mutated Hag construct 10.9, lacking most of this region (see Fig. 3), binds to NCIH292 and middle ear cells (columns N and H in Fig. 3). In addition, *E. coli *carrying the plasmid pBBHS10.32, which specifies the entire A549 binding domain (Fig. 3), does not attach to NCIH292 or HMEE cells (columns N and H in Fig. 3). These results suggest that the portion of Hag important for adherence to middle ear and NCIH292 cells is different from that specifying attachment to A549 pneumocytes. Whether the mutated Hag constructs 10.32 or 10.9 mediate adherence to A549 cells was not tested since we previously demonstrated that Hag expression is not sufficient to confer recombinant bacteria with the ability to attach to this particular cell line. Interestingly, the serum of COPD patients recovering from *M. catarrhalis *infections contain elevated antibody titers against MID residues 367–774 [46], which correspond to aa 355–714 of O35E-Hag. These observations support the hypothesis that the NCIH292 and HMEE epithelial cell binding region of Hag has immunogenic potential, which is a desirable characteristic of a vaccine candidate.

*M. catarrhalis *binds to extracellular matrix proteins including fibronectin [27], vitronectin [28], and laminin [18]; to our knowledge, the organism has not previously been reported to bind to collagen. Our data demonstrate that *E. coli *expressing the *hag *gene product of three *M. catarrhalis *WT isolates (i.e. O35E, V1171, and O12E) gained the ability to attach to type IV collagen (Fig. 2C). *M. catarrhalis *isolates expressing Hag, however, were found to bind poorly to collagen (data not shown), at least when grown under the conditions we used. One possible explanation for these conflicting results is that *M. catarrhalis *cells express an additional surface molecule (not present in the *E. coli *background) that precludes Hag-mediated binding to collagen. This hypothesis is supported by reports demonstrating that expression of certain surface structures can adversely affect the biological function of others. For instance, Pearson and colleagues recently showed that Hag interferes with UspA1-dependent biofilm development [15]. Similarly, the expression of type 1 fimbriae prevents Ag43-mediated autoaggregation of *E. coli *cells, even though Ag43 is still expressed at WT levels on the bacterial surface [53]. Other *E. coli *examples include capsular polysaccharides blocking the function of Ag43 [54], and the autoaggregation proteins TibA as well as AIDA-I functioning as surface antagonists of flagellar motility [55]. Whether *M. catarrhalis *expresses a molecule inhibiting Hag-dependent adherence to collagen remains to be determined. Another possible explanation for these conflicting results is that Hag is expressed at greater levels in *E. coli *compared to *M. catarrhalis*. The biological relevance of *M. catarrhalis *binding to collagen is currently unknown. The molecule is a major component of the extracellular matrix that supports most tissues and type IV collagen is found in the basal lamina, which is the layer on which the epithelium sits. Hag-mediated binding to collagen may therefore facilitate adherence of *M. catarrhalis *to damaged tissues during infection.

## Conclusion

Hag expression is important for adherence to several human epithelial cells relevant to pathogenesis by *M. catarrhalis*, including middle ear, lung and conjunctival cells. This Oca-family adhesin directly mediates binding to type IV collagen as well as middle ear and NCIH292 lung cells, but its expression is not sufficient to confer recombinant bacteria with the ability to attach to Chang conjunctival monolayers. The collagen and epithelial cell binding properties of Hag are separable and residues 385–705 appear to specify the epithelial cell binding domain of the protein. This portion of Hag is different from that proposed by other investigators to be crucial for attachment to A549 cells (i.e. 715–863). Furthermore, conserved structural features shown to specify the adhesive properties of the Oca adhesin YadA (i.e. β-roll), are not important for adherence mediated by Hag. These observations suggest that Hag employs different mechanisms to confer adherence to epithelial cells and collagen.

## Methods

### Bacterial strains, plasmids, tissue culture cell lines, and growth conditions

NCIH292, Chang and HMEE cells were cultured as previously described [23,26,30]. Table 1 lists the bacterial strains and plasmids used in this study. *M. catarrhalis *was grown on Todd-Hewitt agar plates as previously reported [26]. Recombinant *E. coli *cells were cultured using Luria-Bertani medium supplemented with chloramphenicol at a concentration of 15 μg/ml (LB+Cm). *E. coli *EPI300 cells were grown overnight in 5 ml of LB+Cm at 37°C with agitation (200 rpm). The next morning, these cultures were transferred to flasks containing 20 ml of fresh LB+Cm supplemented with 250 μl of Epicentre's 1000 × Copy Control Induction Solution and grown at 37°C with vigorous shaking (300 rpm) for 2 hr (for adherence assays and proteinase K treatment experiments) or 5 hr (for purification of plasmid DNA and protein preparations). To purify the recombinant protein His.Hag.CT (see below), *E. coli *TUNER cells carrying plasmid pBBCT.77 were cultured overnight in 5 ml of LB+Cm supplemented with glucose at a concentration of 0.2%. The next morning, these cultures were transferred to 20 ml of fresh LB+Cm+0.2% glucose and incubated with shaking at 37°C for 1 hr. Protein expression was then induced by adding 25 μl of a 1 M IPTG solution and incubating the cultures with vigorous shaking for 5 hr at 37°C.

**Table 1:** Strains and plasmids

**Strain or plasmid**	**Description**	**Source or reference**
*M. catarrhalis*		
		[26]
O35E	Wild-type isolate	[26]
O35E.TN2	Isogenic *hag *mutant strain of O35E	[26]
O12E	Wild-type isolate	[26]
O12E.Hag	Isogenic *hag *mutant strain of O12E	[26]
TTA37	Wild-type isolate	[26]
TTA37.Hag	Isogenic *hag *mutant strain of TTA37	[26]
V1171	Wild-type isolate	[26]
V1171.Hag	Isogenic *hag *mutant strain of V1171	[26]
McGHS1	Wild-type isolate	[26]
McGHS1.Hag	Isogenic *hag *mutant strain of McGHS1	[26]
*E. coli*		
EPI300	Cloning strain	Epicentre
TUNER	Expression strain	Novagen
Plasmids		
pETcoco-1	Protein expression vector, Cm^r^	Novagen
pBBCT.77	pETcoco-1 expressing O35E-Hag aa 1358–1964 joined to six N-terminal histidine residues, Cm^r^	This study
pCC1.3	Adherence negative plasmid control	[48]
pCC1	Cloning vector, Cm^r^	Epicentre
pELO35.Hag	pCC1 expressing the entire O35E *hag *gene product, Cm^r^	[26]
pBBO12.Hag	pCC1 expressing the entire O12E *hag *gene product, Cm^r^	[26]
pSV1171.Hag	pCC1 expressing the entire V1171 *hag *gene product, Cm^r^	[26]
pBBHS2.24	Deletion derivative of pELO35.Hag, missing aa 71–283	This study
pBBHS3.20	Deletion derivative of pELO35.Hag, missing aa 71–384	This study
pBBHS8.18	Deletion derivative of pELO35.Hag, missing aa 71–384 and 1546–1707	This study
pBBHS6.22	Deletion derivative of pELO35.Hag, missing aa 436–1538	This study
pBBHS5.12	Deletion derivative of pELO35.Hag, missing aa 71–283 and 919–1538	This study
pBBHS10.9	Deletion derivative of pELO35.Hag, missing aa 71–384, 746–1193 and 1546–1707	This study
pBBHS10.32	Deletion derivative of pELO35.Hag, missing aa 71–705 and 1546–1707	This study

### Recombinant DNA methodology

Plasmid DNA was purified from recombinant *E. coli *EPI300 cells using Qiagen's Qiaprep Spin Miniprep Kit with minor modifications to the manufacturer's instructions: two additional washes with the PE buffer were performed and plasmid DNA was eluted from the columns using 50 μl and 30 μl of elution buffer, consecutively. Details of the other standard molecular biology techniques used in this study can be found elsewhere [23,26,30,56].

### Construction of in-frame deletions within *hag*

Primer sewing was used to introduce deletions into the *hag *ORF of *M. catarrhalis *strain O35E that is contained by plasmid pELO35.Hag. Briefly, a 42-mer oligonucleotide was designed such that the 5'-half specifies the region directly upstream of the area to be removed and the 3'-half corresponds to the DNA immediately downstream of the desired deletion. This oligonucleotide, along with a primer corresponding to its complement, was then used to perform inverse PCR with the Stratagene QuickChange^® ^II XL Site-directed Mutagenesis Kit per the manufacturer's recommended guidelines. This PCR reaction was restricted with *Dpn*I to remove the intact WT plasmid DNA template, purified and electroporated into *E. coli *EPI300 cells. These bacteria were allowed to recover for 1 hr at 37°C in 1 ml of LB medium and spread onto LB+Cm agar plates. Chloramphenicol resistant colonies were screened by colony PCR to identify clones containing the intended deletion. Colonies were patched onto LB+Cm plates and placed into PCR tubes. These tubes were microwaved on high-power for 2 min and PCR was performed using Invitrogen's *Taq *DNA polymerase and *hag*-specific primers flanking the deleted region of *hag*. This overall strategy was used to generate plasmids pBBHS2.24, pBBHS3.20, pBBHS5.12, pBBHS6.22, pBBHS8.18, pBBHS10.9, and pBBHS10.32 (Table 1, Fig. 3). All plasmids were sequenced to verify that they contain only the intended in-frame deletions. Construct 5.12 was found to contain an aa substitution at residue 1541 (G^1541 ^→ V). This mutation does not, however, affect our conclusions that residues 385–705 are important for adherence to epithelial cells and that aa 706–1194 are involved in collagen-binding. This belief is based on the phenotypes of constructs 8.18, 10.9 and 10.32 (see Fig. 3), which do not contain this particular mutation or any other aa substitution.

### Production of antibodies against the *M. catarrhalis *strain O35E-Hag protein

A PCR product corresponding to nt 4072–5892 of the O35E-*hag *ORF was amplified from chromosomal DNA with the Invitrogen Platinum *Pfx *DNA polymerase using the oligonucleotide primers HagFQ1358*Asc*I (5'-TTG GCG CGC CTT CAA ATG AAT GTC AAA TCA-3', *Asc*I site underlined) and HagRF1964*Pac*I (5'-CCT TAA TTA ACC AAA GTG AAA ACC TGC ACC-3'; *Pac*I site underlined). The amplicon was purified with Epicentre's PCR Precipitation Solution, digested with *Asc*I and *Pac*I, and ligated into the cognate sites of the plasmid vector pETcoco-1 (Novagen) using T4 DNA ligase (NEB). This ligation mixture was introduced into *E. coli *EPI300 cells by electroporation and chloramphenicol resistant transformants were screened by colony PCR as described above using primers that anneal to regions of pETcoco-1 flanking the cloned DNA insert. This approach yielded the plasmid pBBCT.77, which was sequenced to verify that no mutations were introduced during PCR as well as to confirm that it specifies a protein corresponding to aa residues 1358–1964 of O35E-Hag fused to six N-terminal histidine residues (encoded by pETcoco-1). This protein was designated His.Hag.CT.

The plasmid pBBCT.77 was introduced into chemically competent *E. coli *TUNER cells (Novagen), and the His.Hag.CT protein was purified from these recombinant bacteria with the BugBuster HT Protein Extraction Reagent and the His-Bind Resin System under the manufacturer's recommended conditions (Novagen). For the production of antibodies, female BALB/c mice were immunized with purified His.Hag.CT emulsified in Freund's adjuvants (complete and incomplete, Fisher Scientific) as reported [29]. Recovered murine serum was demonstrated to recognize Hag in western blot experiments using whole cell lysates of the WT strain O35E and its *hag *mutant O35E.TN2 (data not shown).

### Protein preparations and western blot analysis

Recombinant *E. coli *EPI300 cells were suspended in 5 ml PBS to an optical density of 300 Klett units, pelleted by centrifugation and resuspended in 5 ml of 10 mM HEPES buffer. Sarkosyl-insoluble outer membrane (OM) proteins were isolated from these suspensions as described [57]. These OM protein preparations were then analyzed by western blot as reported [26], using murine serum against His.Hag.CT. In some experiments, the Hag-specific monoclonal antibody 5D2 [13] was used in lieu of murine sera against His.Hag.CT. The preparation of whole cell lysates from *M. catarrhalis *and *E. coli *has been described elsewhere [58,59]. Equivalent loads of protein were analyzed in all experiments.

For the treatment of intact bacteria with Proteinase K [60], recombinant *E. coli *cells were suspended to an optical density of 300 Klett units (10^9 ^CFU/ml) in 5 ml of 200 mM Tris-Acetate buffer (pH 8.2) supplemented with 20 mM MgSO_4_. Proteinase K (Novagen) was added to 0.5 ml of bacterial suspensions at a final concentration of 0.5–1.2 μg/ml, and these mixtures were incubated on ice for 15 min. Bacteria were next pelleted and resuspended in ~100 μl PBS supplemented with EDTA-free Protease Inhibitor Cocktail (Roche Applied Science) at a final concentration of 2 ×. SDS-PAGE sample loading buffer was then added to each suspension and they were analyzed by western blot using Hag-reactive antibodies (see above) or with the TonB-specific monoclonal antibody 4F1 [36].

### Adherence assays

Adherence to epithelial cells was measured using a viable cell count attachment assay previously described by our laboratory [23,26,30,48]. For assays involving *M. catarrhalis*, bacteria were incubated with epithelial cells for 5 min prior to washing off unbound bacteria. For assays with *E. coli*, infected monolayers were incubated for 1 hr before removing non-adherent recombinant bacteria. Duplicate attachment assays were performed on at least 3 separate occasions and the results are expressed as the mean (± standard error) percentage of inoculated bacteria that adhered to epithelial cells. Binding of *E. coli *strains to collagen was measured using a modification of this attachment assay. Recombinant *E. coli *cells were suspended in 5 ml of PBS supplemented with 0.15% gelatin (PBSG) to an optical density of 230 Klett units, and 25 μl of each suspension (~10^7 ^CFU) were added to duplicate wells of a 24-well BioCoat murine type-IV collagen tissue culture plate (BD Biosciences) containing 0.5 ml tissue culture medium without antibiotics. Dilutions of each bacterial suspension were spread onto LB agar plates and grown overnight to enumerate the number of CFU used to infect these collagen-coated wells. The inoculated tissue culture plate was centrifuged at 165 × g for 5 min and placed in a 37°C incubator with 7.5% CO_2 _for 1 hr. Each infected well was then washed five times with 0.5 ml PBSG, fixed with 0.5 ml methanol and stained for 15 min with 0.5 ml of a solution containing 0.035% crystal violet and 5% methanol. Wells were washed three times with deionized H_2_O, dried, and photographed on either side of a point in the center of the well (2 fields/well). The number of bacterial cells per field was counted by two individuals and each strain was tested on at least 2 separate occasions. The results are expressed as the mean number of bacterial cells (± standard error) per field normalized by the number of CFU used to inoculate the collagen-coated wells.

### Sequence and statistical analyses

Plasmids were sequenced at the University of Michigan sequencing core. The resulting chromatograms were assembled using the ChromaTool software (BioTools, Inc.), and sequence analysis was performed using Vector NTI 10.1.1 (Invitrogen). Statistical analyses were performed using the Mann-Whitney test provided by the GraphPad Prism Software v. 4.0 (GraphPad). *P *values less than 0.05 were considered statistically significant.

## Authors' contributions

BB and ERL contributed equally to the design of experiments, interpretation of results and writing of the manuscript. BB was responsible for performing most experiments. SL performed some of the adherence assays with *Moraxella catarrhalis *strains.
